# Influence of margin design and restorative material on the stress distribution of endocrowns: a 3D finite element analysis

**DOI:** 10.1186/s12903-022-02063-y

**Published:** 2022-02-05

**Authors:** Ziting Zheng, Jieli Sun, Lifang Jiang, Yuan Wu, Jiahui He, Wenhao Ruan, Wenjuan Yan

**Affiliations:** 1 Department of Stomatology, Nanfang Hospital, Southern Medical University, Guangzhou, China; 2 Department of Stomatology, Beijing Luhe Hospital, Capital Medical University, Beijing, China

**Keywords:** Endocrown, Endodontically treated teeth, 3D finite element analysis, Ceramic, Composite resin

## Abstract

**Background:**

This study aimed to evaluate the stress distributions in endocrown restorations as applied to endodontically treated teeth (ETT), according to the factors of “margin design” (four levels) and “restorative material” (six levels).

**Methods:**

Four 3D-finite elements models were constructed for endocrown restored molars considering different margin designs. Model A was prepared with a flat butt joint margin and received an endocrown with a 2.0-mm occlusal thickness. Model B was prepared with a 20° bevel margin and received an endocrown with a 2.0-mm occlusal thickness. Model C was prepared with an axial reduction and 1-mm shoulder margin and received an endocrown with a 2.0-mm occlusal thickness. Model D was prepared with an anatomic margin and received an endocrown with a 2.0-mm occlusal thickness. The following endocrown materials were used: In-Ceram Zirconia (Zr), Vita Suprinity (VS), IPS Empress (IE), Grandio blocs (GR), VisCalor bulk (VS), and CopraPeek Light (CP). The Load application (600 N) was performed at the food bolus and tooth surface during the closing phase of the chewing cycle. The results for the endocrown and tooth remnants were determined according to the von Mises stress. The failure risk of the cement layer was also calculated based on the normal stress criterion.

**Results:**

Model D (with an anatomic margin) showed the greatest stress concentrations, especially in the irregular and sharp angles of the restoration and tooth remnants. The stress concentrated on the dentin was significantly lower in Model B with a 20° bevel margin (20.86 MPa), i.e., 1.3 times lower than the other three margin designs (27.80 MPa). Restorative materials with higher elastic moduli present higher stress concentrations inside the endocrown and transmit less stress to the cement layer, resulting in lower bonding failure risks. In contrast, materials with an elastic modulus similar to that of dentin presented with a more homogeneous stress distribution on the whole structure.

**Conclusions:**

An endocrown with a 20° bevel margin design could be a favorable preparation option for ETT. Composite resins (GR and VC) exhibit a more even stress distribution, and seem to be more promising materials for endocrown molars.

## Introduction

The decision regarding how to restore endodontically treated teeth (ETT) with extensive coronal loss remains a clinical challenge [1, 2]. Although satisfactory results have been achieved with crowns supported on post and cores over the years, this process has been revealed to weaken the mechanical resistance of the tooth structure, and to increase the incidence of root fracture [3].

With the emphasis on minimally invasive concepts and progress made in adhesive dentistry, endocrown restorations have been introduced as an alternative option for rehabilitating ETT [4, 5]. An endocrown restoration is a monolithic restoration that utilizes the pulp chamber and remaining coronal tooth structure as a means of retention [6]. Its advantages include sealing the root canal and preventing the risk of recontamination. Endocrowns have been reported to provide sufficient intention stability and greater fracture resistance [7–9]. Furthermore, clinical studies are available for ETT restored by endocrowns, and show acceptable clinical performance and longevity [10]. Therefore, endocrown restorations have gained higher satisfaction and attention from clinicians and patients.

Studies have shown that margin forms have significant influences on the biomechanical behavior of ETT restored with posts or crowns [11–13]. Whether there is a similar effect from margin design in endocrown restoration is a point for further investigation. Usually, endocrown is designed with a butt joint margin [14]; However, limited scientific evidence is available to support this recommendation. Recent studies found that a ferrule design could provide greater fracture resistance and allow for fewer catastrophic failure modes for teeth restored by endocrowns [15, 16]. Others have suggested an anatomical margin design with the advantage of the maximum preservation of the tooth structure and morphology [17]. To date, relatively few studies are available to confirm which margin designs of endocrown are more effective for restoring ETT.

Endocrowns are commonly fabricated using ceramic based on leucite ceramics, lithium disilicate, and zirconia. Although ceramics show excellent mechanical properties, they are prone to non-repairable fractures extending to the root, owing to their brittle characteristics [18, 19]. Given this, alternative materials with a more compliant behavior have been introduced for endocrown fabrication, such as resin composites and polymer-infiltrated ceramics. They exhibit higher resilience and more resistance to higher occlusal forces [20, 21]. Recently, a modified polyetheretherketone (PEEK) material, with favorable biocompatibility and good adhesion properties to tooth structures, also become a viable option for endocrowns [22]. Recent studies have also demonstrated that endocrowns prepared with distinct restorative materials using a direct technique are more tooth-friendly than ceramic endocrowns, resulting in less aggressive failures [6, 23]. Thus, the possibility of using materials with elastic moduli from 3.7 to 200 GPa raises the question of how these restorations will behave mechanically. Understanding the mechanical behaviors of the different materials used in the fabrication of endocrowns is important.

Finite element analysis (FEA) has been used in dentistry to evaluate the stress distributions generated by masticatory loads, owing to its standardization and effectiveness. It can detect stress concentration regions where failures may occur. Usually, the failure origin consists of points of greater stress concentrations, as previously confirmed by FEAs [19, 24–27]. Therefore, the purpose of this study was to evaluate the influences of the margin design and material type on the biomechanical behaviors of endocrowns for restoring ETT using FEA. The null hypotheses were that the margin design and type of restorative material would not interfere in the stress distribution of the endocrown restoration.

## Materials and methods

This work was approved by the ethics committee of Nanfang Hospital, Southern Medical University, Guangzhou, PR China (NFEC-2017-141). An intact extracted mandibular first molar was scanned using microcomputed tomographic imaging (Quantum GX; PerkinElmer). The obtained data were imported into an interactive medical image control system (Mimics 15.0; Materialise NV, Belgium) in a “Digital Imaging and Communications in Medicine” format. The three-point clouds (enamel, dentin, and pulp) were separated according to the different pixel densities. The contour for each portion was then generated using software (Geomagic Studio; Geomagic Inc). A three-dimensional solid model was then reconstructed with a computer-aided design software program (SolidWorks 2014; Dassault Systèmes). To simulate an endodontically treated molar, the pulp in the root canal was replaced with gutta percha, and a flowable resin (SDR; Dentsply Sirona) was used to fill the pulp chamber floor [27].

**Fig. 1:**
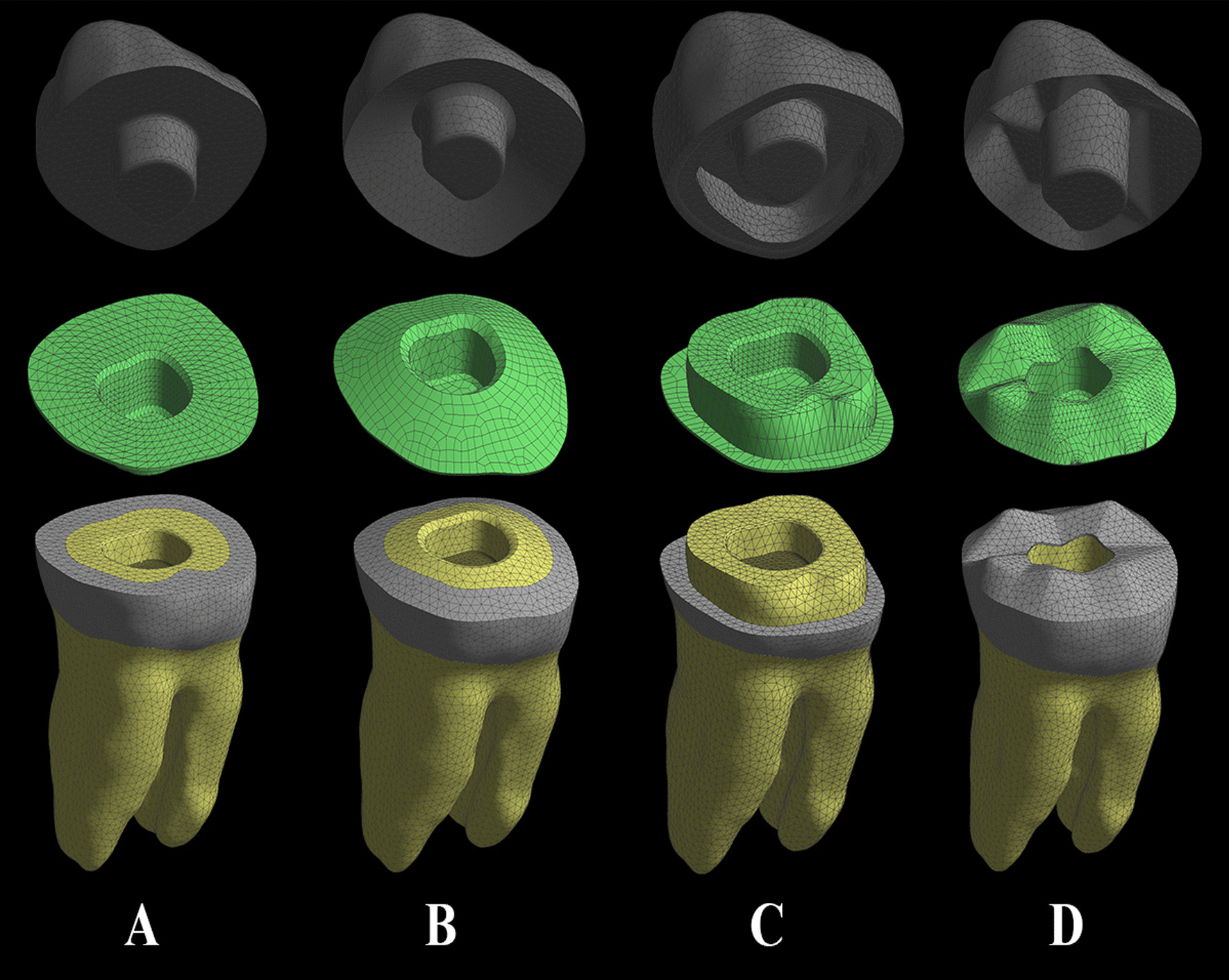
Finite element models of endodontically treated mandibular molar restored with endocrown using four margin designs. **A** Flat butt joint margin. **B** A 20° bevel margin. **C** An axial reduction and 1 mm shoulder margin. **D** Anatomic occlusal preparation margin

Stating from the endodontically treated molar model, four endocrown restored models with different margin designs were created (Fig. 1). Model A was prepared with a flat butt joint margin, and received an endocrown with a 2.0-mm occlusal thickness (Fig. 1A); Model B was prepared with a 20° bevel margin and received an endocrown with a 2.0-mm occlusal thickness (Fig. 1B); Model C was prepared with an axial reduction and 1-mm shoulder finish line and received an endocrown with a 2.0-mm occlusal thickness (Fig. 1C); Model D was prepared with anatomic reduction for all cusps, and received an endocrown with a 2.0-mm occlusal thickness (Fig. 1D). All endocrowns were prepared with a 2.0-mm intracoronal extension with an 8° wall inclination angle [28]. The cement layer was modeled with a 120-μm thickness between the internal surfaces of the restoration and bonding surfaces of the teeth [24, 29]. The four endocrown-restored models were duplicated and then restored using six restorative materials, including zirconia ceramic (In-Ceram Zirconia, Zr; Vita Zahnfabrik), zirconia-reinforced glass–ceramic (Vita Suprinity, VS; Vita Zahnfabrik), high-leucite content ceramic (IPS Empress, IE; Ivoclar-Vivadent AG), composite resin (Grandio blocs, GR; VOCO), termoviscous bulk-fill composite (VisCalor bulk, VS; VOCO), and PEEK (CopraPeek Light, CP; Whitepeaks).

The geometries were imported in the “Standard for the Exchange of Product Data” format for the ANSYS software CAE (Ansys 20.0; Swanson Analysis Inc); in the software, they were divided into meshes composed of nodes and tetrahedral elements. A convergence test of 10% mesh control was used to determine the number of elements and nodes to generate four models, as listed in Table 1. The mechanical properties of the dental structures and materials were determined from published values (Table 2). All structures were assumed to be linearly elastic, isotropic, and homogenously distributed. All contacts were ideally cast.

**Table 1:** Number of elements and nodes of four models

Model	Description	Elements	Nodes
A	A flat butt joint margin	226,159	344,603
B	A 20° bevel margin	227,229	347,542
C	An axial reduction and 1 mm shoulder margin	228,973	351,462
D	A 2-mm anatomic occlusal preparation margin	229,480	352,005

**Table 2:** Material properties

Material	Elastic modulus (GPa)	Poisson’s ratio	Tensile strength (MPa)	Adhesive bond strength to dentin (MPa)
Enamel [30]	84.10	0.33		
Dentin [30]	18.60	0.31		
Gutta percha [20]	0.69 (× 10^–3^)	0.45		
Periodontal ligament [20]	0.07	0.45		
Spongious Bone [20]	1.37	0.30		
Cortical Bone [20]	13.70	0.30		
In-Ceram Zirconia [24]	200.00	0.31		
Vita Suprinity [20]	104.90	0.21		
IPS Empress [24]	65.50	0.20		
Grandio blocs [20]	18.00	0.26		
VisCalor bulk^a^	12.30	0.28		
CopraPeek Light [31]	3.70	0.40		
Flowable resin [20]	7.00	0.25		
Resin cement [29]	7.40	0.35	51.90^a^	33.80^a^

^a^Supplied by the manufacturer

**Fig. 2:**
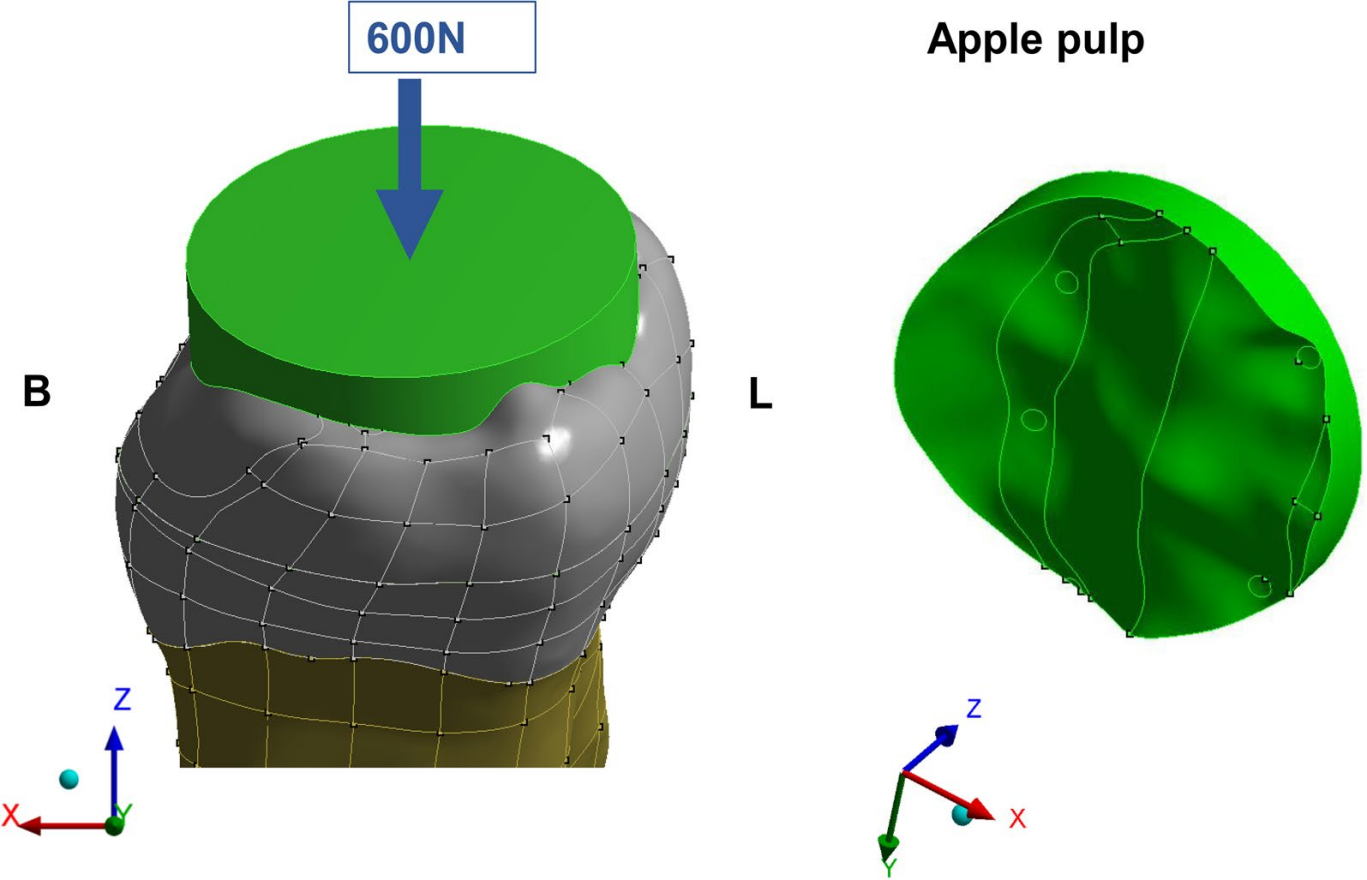
Food modeling on the occlusal surfaces

The load application (600 N) occurred similar to the methodology described in previous studies [25, 29, 30], and considered the contact between the food bolus and tooth surface during the closing phase of the chewing cycle (Fig. 2). Solid food (apple pulp), with an elastic modulus of 10 MPa and Poisson’s ratio of 0.3, was modeled onto the restoration’s occlusal surface. Slide-type contact elements were used between the tooth surface and food, as shown in Fig. 2. In the boundary condition, the fixation was applied at the base of the bone tissue and was fixed with zero nodal displacements.

For the restoration and dental structures, the von Mises stress (VMS) was calculated. For the cement layer, the normal stress perpendicular to the insertion trajectory (X-axis) was recorded. In addition, the cohesive and adhesive failure risks for the cement layer were calculated as the stress peak value / tensile strength, and, stress peak value / adhesive bond strength to dentin, respectively [25, 29, 32].

## Results

**Fig. 3:**
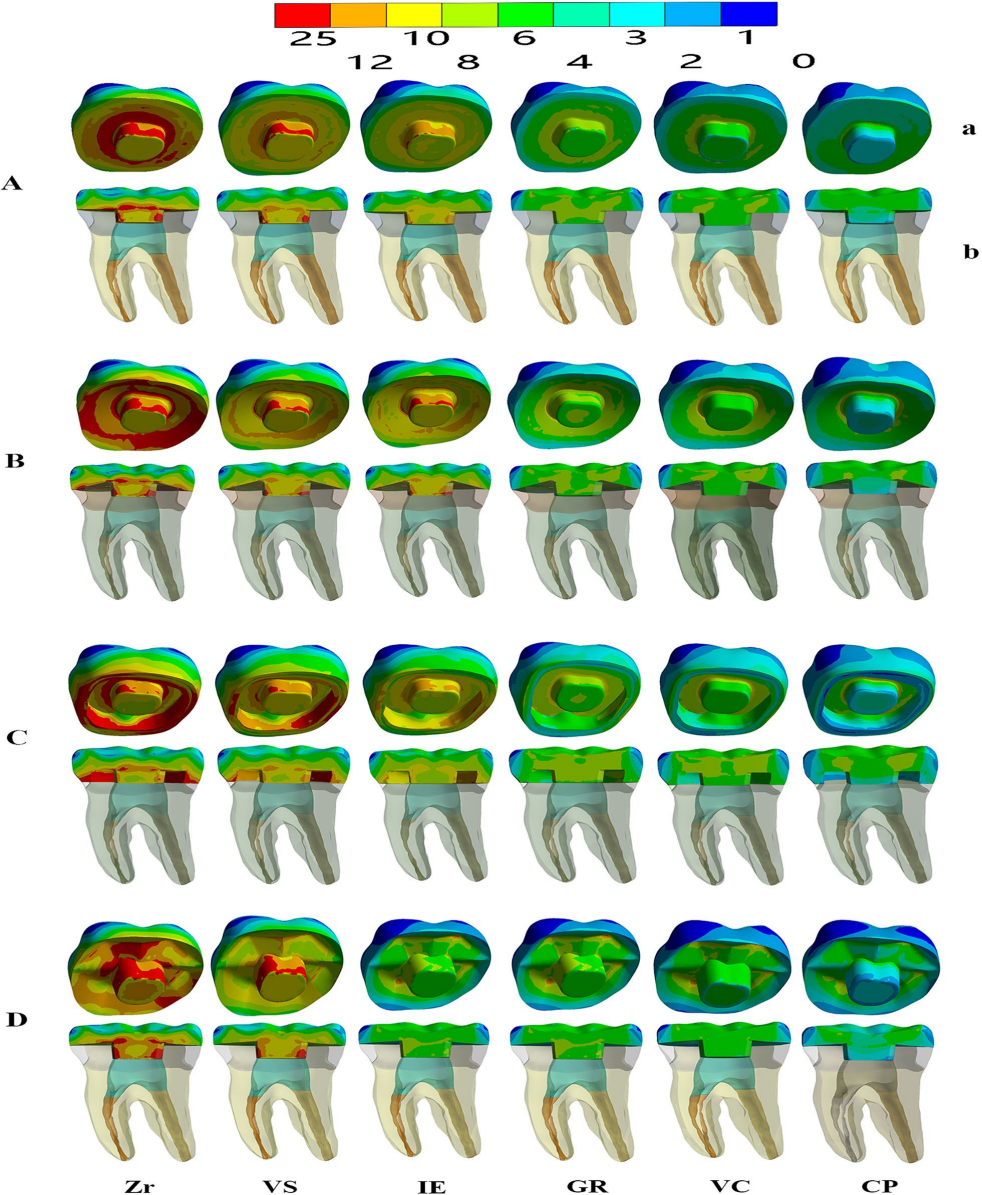
Stress distribution (MPa) in endocrown restorations under loading according to the margin design (**A**–**D**) and restorative materials (Zr, VS, IE, GR, VC, and CP). **A** Flat butt joint margin; **B** a 20° bevel margin; **C** an axial reduction and 1 mm shoulder margin; **D** anatomic occlusal preparation margin. a, Restoration; b, Restoration in the sagittal plane. Zr, Zirconia; VS, Vita Suprinity; IE, IPS Empress; GR, Grandio blocs; VC, VisCalor bulk; CP, CopraPeek Light

**Fig. 4:**
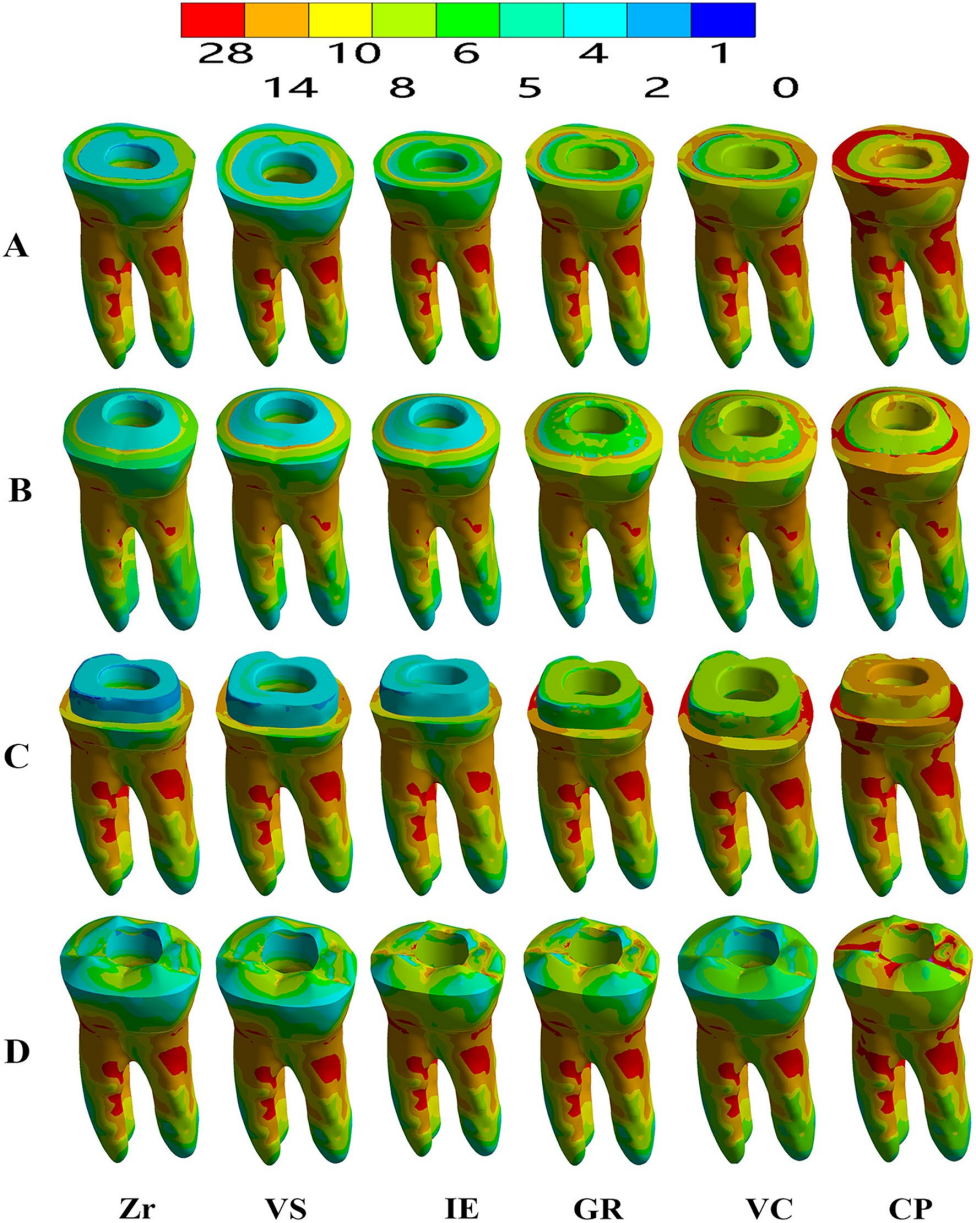
Stress distribution (MPa) in dental remnant structure under loading according to the margin design (**A**–**D**) and restorative materials (Zr, VS, IE, GR, VC, and CP). **A** Flat butt joint margin; **B** a 20° bevel margin; **C** an axial reduction and 1 mm shoulder margin; **D** anatomic occlusal preparation margin. Zr, Zirconia; VS, Vita Suprinity; IE, IPS Empress; GR, Grandio blocs; VC, VisCalor bulk; CP, CopraPeek Light

**Fig. 5:**
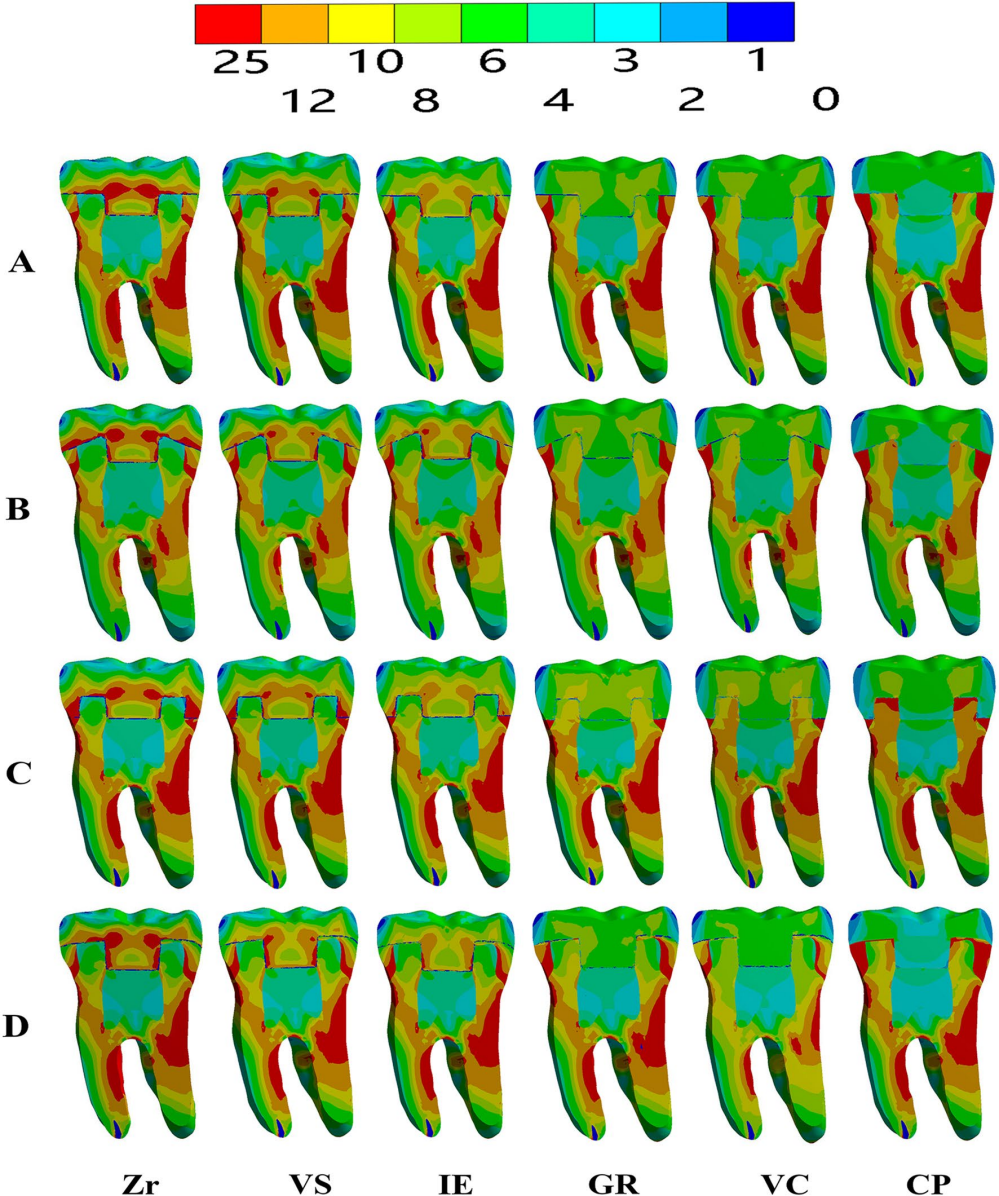
Stress distribution (MPa) in overall structures under loading according to the margin design (**A**–**D**) and restorative materials (Zr, VS, IE, GR, VC, and CP). **A** Flat butt joint margin; **B** a 20° bevel margin; **C** an axial reduction and 1 mm shoulder margin; **D** anatomic occlusal preparation margin. Zr, Zirconia; VS, Vita Suprinity; IE, IPS Empress; GR, Grandio blocs; VC, VisCalor bulk; CP, CopraPeek Light

The VMS distribution results were obtained for the endocrown (intaglio surface and sagittal cut) and dental remnant structure through colorimetric graphs (Figs. 3, 4, 5). When using Zr as the restorative material, in the flat butt margin model, the stress was mainly concentrated on the intaglio surface of the endocrown, especially in the axial walls and cavity floor (Fig. 3), the mesial and distal sides of the cervical region of the enamel (Figs. 4, 5), and the furcation area and distal side of the dentin (Fig. 5). In the 20° bevel margin model, the regions where the VMS was concentrated in the restoration and enamel were similar to those in the flat butt margin model. However, the stress was significantly decreased in the dentin (Table 3). The highest VMS in the rounded shoulder margin model was concentrated around the distal-mesial margin of the enamel, axial walls and shoulder margin of the endocrown. For the anatomic margin model, the stress accumulated in the irregular and sharp angles of the restoration and tooth remnants.

**Table 3:** Group distribution according to the margin forms and restorative material type of the von Mises stress (MPa) obtained in restoration and tooth remnants

Model	Material	Restoration	Enamel	Dentin
A	Zr	26.19	17.75	27.80
	VS	24.43	17.83	27.80
	IE	21.89	17.93	27.79
	GR	11.36	18.38	27.79
	VC	8.94	16.43	27.79
	CP	8.46	26.40	27.78
B	Zr	16.85	16.43	20.86
	VS	17.35	16.53	20.86
	IE	16.85	16.54	20.86
	GR	9.29	18.04	20.86
	VC	9.53	18.44	20.86
	CP	8.47	21.94	20.86
C	Zr	20.61	15.83	27.91
	VS	17.62	15.70	27.90
	IE	15.77	16.29	27.89
	GR	10.20	20.34	27.89
	VC	10.27	21.53	27.89
	CP	8.49	25.66	27.89
D	Zr	27.94	18.00	27.83
	VS	26.06	18.12	27.83
	IE	22.11	20.15	27.83
	GR	12.61	37.95	27.82
	VC	12.78	40.27	27.82
	CP	8.69	54.04	27.01

A, flat butt joint margin; B, a 20° bevel margin; C, an axial reduction and 1 mm shoulder margin; D, anatomic occlusal preparation margin. Zr, In-Ceram Zirconia; VS, Vita Suprinity; IE, IPS Empress; GR, Grandio blocs; VC, VisCalor bulk; CP, CopraPeek Light

Regardless of the margin types of restoration, when using restorative materials (VS, IE, GR) with a lower elastic modulus than Zr, the VMS remained concentrated in these parts but increased both in the enamel and in the pulp cavity walls of the dentin, and decreased in the restoration (Figs. 3, 4, 5). From the sagittal plane of the overall structures (Fig. 5), it can be seen that GR and VC exhibited a more homogeneous stress distribution. However, for the PEEK (with the elastic modulus much lower than dentin), a greater amount of stress was concentrated on the remnant structures (Fig. 5).

For the cement line, all of the simulated models showed a higher adhesive failure risk (0.14–1.30) in comparison to cohesive failure risk (0.09–0.846) (Table 4). In the Zr endocrown, the anatomic margin model presented the highest failure risk (7.3%), at 2.70 times greater than the lowest risk in the 20° bevel margin model (2.7%). For the other restorative materials, the failure risks increased in all models and followed a similar trend to that of Zr. Accordingly, it can be seen that the stress peaks and failure risks were decreased with the increase of elastic modulus of the restorative materials used in endocrown for the normal stress criteria.

**Table 4:** The normal stress peak (MPa), failure risk of cohesive and adhesive for cement layer

Model	Material	Stress peak	Failure risk	
			Cohesive	Cohesive
A	Zr	0.55	0.011	0.016
	VS	0.71	0.014	0.021
	IE	0.88	0.017	0.026
	GR	1.42	0.027	0.042
	VC	1.63	0.031	0.048
	CP	3.14	0.061	0.093
B	Zr	0.47	0.009	0.014
	VS	0.57	0.011	0.017
	IE	0.59	0.011	0.017
	GR	1.10	0.022	0.033
	VC	1.24	0.024	0.037
	CP	2.43	0.047	0.072
C	Zr	0.57	0.011	0.017
	VS	0.75	0.014	0.022
	IE	0.90	0.017	0.027
	GR	1.72	0.033	0.051
	VC	1.98	0.038	0.059
	CP	4.24	0.082	0.125
D	Zr	1.48	0.029	0.044
	VS	3.02	0.058	0.089
	IE	4.15	0.080	0.123
	GR	6.66	0.128	0.197
	VC	6.85	0.132	0.203
	CP	43.93	0.846	1.300

A, flat butt joint margin; B, a 20° bevel margin; C, an axial reduction and 1 mm shoulder margin; D, anatomic occlusal preparation margin. Zr, In-Ceram Zirconia; VS, Vita Suprinity; IE, IPS Empress; GR, Grandio blocs; VC, VisCalor bulk; CP, CopraPeek Light

## Discussion

As an adhesive and conservative coronal restoration, the clinical performance and durability of endocrown not only depend on the available design parameters but are also on the mechanical properties of the restorative materials. In this study, we evaluate the stress distribution of ETT restored with endocrown restorations according to the factors “margin design” (four levels) and “restorative material” (six levels). The results demonstrated that both factors significantly influenced the biomechanical behavior as a function of the endocrown restorations, thus rejecting the null hypotheses.

The stress distribution patterns depend strongly on the functional loads in the FEA study. Previous studies applied an axial load to the occlusal surface based on the tripoidism concept (considered as function contacts) [24, 33–35], resulting in the stress of the restoration always being concentrated on the loading point. In practice, the functional contacts during the chewing cycle are regional, rather than individual points. Furthermore, mastication in oral conditions is a motor activity and can be affected by the texture of the food bolus [36]. Consequently, in this study, the food bolus was applied on the surface of an endocrown that could uniformly distribute the loading for better simulating the chewing process [25, 30, 37].

From a minimally invasive standpoint, the anatomic occlusal preparation margin shows the maximum preservation of the tooth structure [38]. However, from the results of the FEA, higher-stress concentrations were observed in the restoration and tooth remnants, particularly occurring in the irregular and sharp angles areas (Figs. 3, 4), making them more susceptible to fracture. For the shoulder margin design, stress was observed in the cavity walls of restoration and the enamel of the cervical region, as well as at the margin interface between the dental tissue and restorations (Fig. 5). This is in accordance with previous studies that proposed the addition of short axial walls with a shoulder finish line could counteract the shear stresses through the walls, and provide a better load distribution through the margin [15, 16]. Nevertheless, in the meantime, owing to such a stress distribution, there is a greater likelihood of fractures occurring at the margin of the endocrown or in the enamel and leakage around the affected restoration.

Considering the clinical operability, endocrowns prepared with a butt joint margin and 20° bevel margin are more efficient and less technique sensitive than those based on the other two margins. Furthermore, in this study, greater uniform stress distributed over the cervical butt joint and axial walls was observed in the butt joint margin and 20° bevel margin (Fig. 5). This suggested that a stable surface could better withstand and disperse the stresses through tooth structure [4, 5, 14]. The 20° bevel margin achieved the lowest VMS peak values in the restoration and remnant structure. Moreover, it presented a more homogeneously stress distribution in the sagittal plane of the overall structures. This implies that a 20° bevel margin might be better at dispersing the compressive and shear force through the margin, moderating the stress concentrates [39], and reducing the risk of future fracture in ETT.

In this study, the influences of the material type on the stress distributions of endocrown-restored ETT were also investigated. According to the results, the greater the elastic modulus of the restorative material, the higher the stress peaks observed in the restoration themselves, regardless of the margin types for the restoration. Zr with the highest elastic modulus (200.0 GPa), achieved the highest stress value, i.e., at least three times greater than the lowest value of CP, with the lowest elastic modulus (3.70 GPa). Considering the observed stress peaks (although it does not reach the fracture strength of Zr), it may be harmful when located in the lower portion and edge area of the endocrown, where it can easily lead to the failure of the restoration. When evaluating the stresses distribution in a sagittal section, it is possible to observe a stress concentration present on the intaglio surface of the endocrowns, validating the results from previous studies, i.e., that this region is where the propagation of a crack in brittle materials begins [20, 35].

According to the literature [24, 29], the cement layer was modeled with a thickness of 120-μm in our study. This layer has gradually attracted increased attention, as the bonding failures of this layer are closely related to the longevity of the adhesive restorations. Referring to Table 4, it can be seen that the more rigid the endocrown, the less stress that reaches the cement layer. Referring to Table 4, it can be seen that the more rigid the endocrown, the less stress that reaches the cement layer. This behavior suggests that the use of materials with higher elastic moduli can minimize the damage to the cement layer, thus decreasing both of the evaluated cement failure risks [24, 32, 40]. Nevertheless, in clinical practice, the cement line is minimal when correct cementation is completed; thus, the differences between the six restorative materials are relatively few, even though they are significant. According to the colored graphs, with a difference of at most 2 MPa (except for the PEEK in group D) with an anatomic margin, it is impossible to predict if they clinically present very different behaviors potentially promoting cement fracture. Despite this limitation, materials with a low elastic modulus allow a notable passage of stress to the cement, and therefore, careful cementation should always be performed. Further studies on the cement layer and the corresponding mechanical performance and survival of the endocrown should be conducted.

Regarding the dental remnant structure, it was observed that materials with much higher or lower elastic modulus than dentin seem to transfer more stress to the pulp chamber and axial walls of the dentin. In the PEEK group, the maximum stress was transferred to the dental remnant structure, probably owing to the deformation beyond the material that could not reduce the force transferred to the tooth as a stress breaker. The GR or VC materials, with an elastic modulus more approaching that of dentin were more flexible and dissipated more energy under the same load conditions, Thus, an endocrown made of GR or VC exhibited a more homogeneous stress distribution, as shown in the sagittal plane (Fig. 5). This suggested that only when restorative materials demonstrate an elastic modulus approaching dentin, do they exhibit better biomechanical behavior and less probability of fracture in dental structures. It is of great importance that all parts involved in the rehabilitation (such as the endocrown and remnant dental element), form a cohesive whole, thereby simulating the properties of the dentin-enamel junction, which is guaranteed to have a long-term survival rate. In terms of homogeneously stress dissipation, we suggest that materials with similar modulus to that of dentin would be more compliant in the endocrown approach.

Previous studies found that the available surface of adhesion increased over 47% by extending the endocrown coverage to the external axial surfaces [15]. However, in this study, no significant difference was observed in the adhesive failure risk between these two margin designs. As shown in Fig. 4, higher stresses were observed in the cervical margin area of the shoulder margin compared with the flat joint margin; this might significantly increase the risks of the debonding of the restoration. In addition, clinical studies have revealed some difficulties during the fabrication of endocrown with shoulder margin, owing to the limited milling area between the pulpal inlay and proximal axial ferrule walls [41]. As such, the butt joint margin presents better protection of the adhesive interface and restoration from detachment.

To the best of our knowledge, this is the first study to fabricate endocrowns using a new-generation bulk-fill composite resin (VC). Compared to conventional composites, VC is composed of a higher percentage of filler content (83%). This may have influenced modulus development within the restoration [42], i.e., leading it to exhibit a more compliant biomechanical behavior in the study. Moreover, VC presents a greater depth of cure and generates less polymerization stress when compared with conventional formulations, thereby, positively influencing the restoration resistance to fatigue [6]. It seems that the use of bulk-fill composite should be considered in the future for the restoration of ETT with the endocrown approach.

Several limitations of this study should be mentioned. The polymerization shrinkage effects of the cement layer were not performed in this FEA. As reported in the literature [33], cement polymerization shrinkage is a centrifugal contraction and may cause a stress concentration in the bonding interface. However, from the FEA results, it seems that polymerization shrinkage of the cement layer has a smaller influence than the endocrown or dental structure for producing stresses, owing to its normal thickness of 120 μm in our study [30]. In addition, this study only analyzed the stress distribution of endocrown molars under a static load at the closing phase of the chewing cycle; this cannot accurately represent actual clinical situations. Future studies should consider details such as the polymerization shrinkage of the resinous materials and the results of dynamic loading. Long-term clinical trials are also needed to support the results of the present in vitro study.

## Conclusions

Considering the limitations of the present study, it can be concluded that endocrown designed with a 20° bevel margin could be the favorable preparation option for endodontically treated teeth. Composite materials (GR and VC) with an elastic module more consistent with dentin, presented a more evenly stress distribution, thus being a promising alternative for the manufacture of endocrown restorations.

## Data Availability

All data generated or analysed during this study are included in this published article [and its supplementary information files].

## Abbreviations

3D: Three dimensional
ETT: Endodontically treated teeth
FEA: Finite element analysis
VMS: Von Mises stress
Zr: In-Ceram Zirconia
VS: Vita Suprinity
IE: IPS Empress
GR: Grandio blocs
VC: VisCalor bulk
PEEK: Polyetheretherketone
CP: CopraPeek Light
